# Resistance to Antibiotics in Thermophilic Campylobacters

**DOI:** 10.3389/fmed.2021.763434

**Published:** 2021-11-11

**Authors:** Ema Aleksić, Biljana Miljković-Selimović, Zoran Tambur, Nikola Aleksić, Vladimir Biočanin, Stevan Avramov

**Affiliations:** ^1^Faculty of Stomatology Pancevo, University Business Academy in Novi Sad, Pančevo, Serbia; ^2^Faculty of Medicine, University of Niš, Niš, Serbia; ^3^Institute for Cardiovascular Disease “Dedinje, ”Belgrade, Serbia; ^4^Institute for Biological Research “Siniša Stanković,” University of Belgrade, Belgrade, Serbia

**Keywords:** campylobacter, antibiotic resistance, mechanisms of resistance, bacterial enterocolitis, significance of resistant strains

## Abstract

*Campylobacter jejuni* (*C. jejuni*) is one of the most frequent causes of bacterial enterocolitis globally. The disease in human is usually self-limiting, but when complications arise antibiotic therapy is required at a time when resistance to antibiotics is increasing worldwide. Mechanisms of antibiotic resistance in bacteria are diverse depending on antibiotic type and usage and include: enzymatic destruction or drug inactivation; alteration of the target enzyme; alteration of cell membrane permeability; alteration of ribosome structure and alteration of the metabolic pathway(s). Resistance of *Campylobacter spp*. to antibiotics, especially fluoroquinolones is now a major public health problem in developed and developing countries. In this review the mechanisms of resistance to fluoroquinolones, macrolides, tetracycline, aminoglycoside and the role of integrons in resistance of *Campylobacter* (especially at the molecular level) are discussed, as well as the mechanisms of resistance to β-lactam antibiotics, sulphonamides and trimethoprim. Multiple drug resistance is an increasing problem for treatment of campylobacter infections and emergence of resistant strains and resistance are important One Health issues.

## Introduction

Antibiotic resistance in bacteria is caused by the following mechanisms: enzymatic destruction or inactivation of the drug, alteration of the target enzyme, alteration of cell membrane permeability, alteration of ribosome structure, and alteration of the metabolic pathway. Resistance of *Campylobacter spp*. to antibiotics has become a major public health problem in recent years, both in developed and developing countries (1–4). In some countries, there has been a trend of increasing resistance to macrolides, the drugs of choice in the treatment of *Campylobacter*-induced enterocolitis. In the USA and Canada, the prevalence of resistance of *C. jejuni* to erythromycin in isolates obtained from humans, broiler chickens, and cattle is up to 10%. High incidence of resistant Campylobacter infections has been confirmed even in infants under 5 years of age (5). In Africa, resistance to erythromycin is pronounced in isolates of human origin, while *C. jejuni* and *C. coli*, isolated in animals, are less resistant. In Asia, the resistance of *C. jejuni* to macrolides is less pronounced, while strains of *C. coli* are more resistant. In Australia, similarly to the findings on other continents, resistance to macrolides has been observed mainly in *C. coli* strains (6). Fluoroquinolone resistance, as well as a rapid increase in the number of isolated resistant strains, have been observed in the United States, Canada, and most European countries (7). In both Africa and Asia, the finding of fluoroquinolone-resistant *Campylobacter* dominates, while in Thailand and Hong Kong, the resistance rate is >80% (6). In Australia and New Zealand, the rate of fluoroquinolone-resistant strains is significantly lower than in other regions (8, 9). A significant percentage of fluoroquinolone-resistant strains is also observed in Serbia (10). Tetracycline resistance in *Campylobacter spp*. is described in many countries, however, there are large geographical differences in the distribution of sensitivity profiles (6). The selection and spread of resistant strains of thermophilic *Campylobacter* are attributed to the uncontrolled use of drugs (especially macrolides) as growth promoters in veterinary medicine, in disease prophylaxis and therapy (11).

## Mechanisms of Antibiotic Resistance

### Mechanisms of Resistance to Fluoroquinolones

The enzyme DNA gyrase is composed of two pairs of subunits, GyrA and GyrB, while topoisomerase IV also consists of two subunits of ParC and ParE (12). Resistance to fluoroquinolones is the result of a change in one or more amino acids in topoisomerase as well as in gyrase. In *Campylobacter* strains, resistance to fluoroquinolones results from a mutation in the gyrA gene encoding a subunit of the GyrA DNA gyrase (13, 14). To this day, no mutations in gyrase B DNA have been observed in *Campylobacter* that would be associated with resistance to fluoroquinolones. The most commonly observed mutation in fluoroquinolone-resistant *Campylobacter* isolates was the point mutation of the Thr-86-Ile in the gyrA gene (15), which in gyrase at position 86 leads to threonine replacement by isoleucine (12). In the genus *Campylobacter*, resistance to fluoroquinolones appears to be due mainly to a mutation in the gyrA gene, which encodes GyrA, a subunit of DNA gyrase (15, 16). In *C. jejuni*, the high level of resistance to ciprofloxacin is due to the point mutation of the Thr-86-Ile in gyrA (17). Another described gyrA mutation in *C. jejuni* includes the Thr-86-Ala (high level of nalidixic acid resistance and low level of ciprofloxacin resistance). The following mutations have also been described: Ala-70-Thr, Thr-86-Lys, Asp-90-Asn, and Pro-104-Ser (15, 17, 18). Double point mutations in gyrA have also been described in which the Thr-86-Ile mutations are combined with Asp-85-Tyr, or Asp-90-Asn, or Pro-104-Ser (15). Concerning the mutation in Asp-90 or Ala-70, mutations in Thr-86 are thought to be associated with a higher level of resistance, which is expressed as the Minimum Inhibitory Concentration (MIC), which with nalidixic acid has a range from 64 to μg/ml, and with ciprofloxacin, from 16 to 64 μg/ml. *C. jejuni* isolates resistant to even higher levels of fluoroquinolones (125 μg/mL MIC ciprofloxacin) were thought to carry two mutations: one at the gyrA at the position of Thr-86 and the other at the parC subunit at Arg-139 (19). However, later research did not confirm a mutation in the topoisomerase gene (20). The Asp90-Asn mutation is associated with a low level of resistance (MIC = 4–8 μg/ml), and the Thr86-Ile mutation in the GyrA gyrase subunit is associated with a high level of resistance (MIC ≥ 16 μg/ml) to ciprofloxacin. The presence of both mutations is thought to lead to high levels of resistance (MIC ≥ 128 μg/ml). The Thr-86-Ile mutation leads to different levels of resistance, which indicates the role of other mechanisms. However, this unique change alone (Thr86-to-Ile) is sufficient to achieve a high level of resistance (MIC up to 256 μg/ml for ciprofloxacin). Although in *C. jejuni* and *C. coli*, a single modification of the GyrA subunit is sufficient to give rise to a fluoroquinolone-resistant phenotype, fluoroquinolone resistance is also due to a decrease in the outer membrane permeability and efflux pump activity (21). In *C. jejuni* and *C. coli* strains, in addition to mutations in GyrA, the efflux pump for several drugs, CmeABC, is also thought to contribute to fluoroquinolone resistance by reducing drug accumulation in *Campylobacter* cells (22, 23). Therefore, CmeABC is thought to act synergistically with mutations in the gyrA gene in the development of fluoroquinolone resistance (6). According to other authors, the efflux pump does not participate or has a very small role in the development of fluoroquinolone resistance (20). To understand the role of efflux transporters for several drugs in *C. jejuni*, Jeon et al. (24) described the action of the MFS transporter (Cj1375) labeled as CmeG. The results indicated that *Campylobacter spp*. and CmeG functioned as an efflux transporter thus contributing to antibiotic resistance, especially to fluoroquinolones, and to defense against oxygen activity (24). A common fluoroquinolone of choice, ciprofloxacin, has been reported in developing countries with levels ranging from 30 to >84%. As with other fluoroquinolones, the frequency of isolating ciprofloxacin-resistant strains of *Campylobacter* is rising (25, 26).

### Mechanisms of Resistance to Macrolides

Erythromycin, a member of the macrolide family of 14 C atoms, and other macrolide antibiotics bind to the 50S subunit of the bacterial ribosome and prevent elongation of the polypeptide chain (27), leading to an early release of peptidyl-tRNA. The sites of action of macrolides are parts of the 23S rRNA subunit and ribosome proteins, L4 and L22 (6, 28). Proteins L4 and L22 form portions of the polypeptide exit channel in the bacterial ribosome 70S and have been described in several bacterial species (12). Resistance to erythromycin may result from enzymatic inactivation of the drug, may be due to targeted modifications of the enzyme due to mutation or methylation, or may be due to active efflux of the drug (29). Resistance to macrolides in *Campylobacter spp*. is mainly associated with target modification or active efflux (11, 30). Modification on the ribosome that results in resistance to macrolides in *Campylobacter* may occur as a result of enzymatic methylation or due to a point mutation in the genes for 23S rRNA and/or ribosome proteins, L4 and L22 (31). The point mutation in the V domain of 23S rRNA, on the other hand, is the most common mechanism of macrolide resistance in *C. jejuni* and *C. coli* (32). This point mutation occurs at positions 2,074 and 2,075 in 23S rRNA. In addition to this mutation, which has been confirmed to lead to resistance, mutations A2074C, A2074G, and A2075G, have been found to result in high levels of resistance to macrolide antibiotics (erythromycin MIC > 128 μg / ml) in *C. jejuni* and *C. coli* (11). In clinical and field isolates, the A2075G mutation is the most commonly observed one (33, 34). *C. coli* and *C. jejuni* possess three copies of the rrn operon (35). The macrolide resistance mutation in *Campylobacter* is typically present in all three copies in the 23S rRNA gene. However, it has been found that some mutations, such as A2074T, which leads to low levels of erythromycin resistance, may not be present in all copies of the 23S rRNA gene (35). Mutations that prevent macrolide binding have also been described in ribosomal proteins, L4 and L22, which form portions of the polypeptide exit tunnel within the bacterial ribosome 70S and which are present in several bacterial species (12). A recent study described 13 isolates of *C. jejuni* and *C. coli* that possessed a characteristic A2075G mutation, as well as one or more substitutions in the L4 protein and two or more substitutions in the L22 protein. The unique substitution of A103V, demonstrated for L22 protein in each of the two isolates of *C. jejuni* and *C. coli*, is thought to lead to a high resistance phenotype (32). Moreover, in macrolide-resistant *C. jejuni* and *C. coli* mutants, which have been described in recent studies, modifications occur in the L4 (G74D) and L22 ribosome proteins (insertion at positions 86 or 98). Also, modifications in L4 and L22 have been observed to contribute to a low level of erythromycin resistance in *C. jejuni* strains (36). In addition to the target modification in *Campylobacter*, active efflux also contributes to resistance to macrolides (25, 33). In isolates with medium or low levels of resistance to macrolides, inactivation of the CmeABC efflux pump completely restores the sensitivity of the isolates (37). Even in highly resistant Campylobacter strains with A2074G or A2075G mutations, CmeABC inactivation also significantly reduces the level of resistance to macrolide antibiotics, indicating that this efflux system functions synergistically with the target mutations (37). It has also been shown that the synergy between the CmeABC efflux pump and mutations in the L4 (G74D) and L22 ribosome proteins (insertions at positions 86 or 98) leads to macrolide resistance in both *C. jejuni* and *C. coli* (30). Resistance to macrolides can occur in two types: resistance to high levels of drug (HLR) (38) and resistance to low levels of resistance (LLR) (39). In HLR, the MIC of erythromycin is higher than 128 mg / L, and in LLR, MICs range from 8 to 16 mg/L (31, 38).

### Mechanisms of Tetracycline Resistance

Tetracyclines (e.g., tetracycline, chlortetracycline, and minocycline) bind to the ribosome and inhibit the placement of aminoacyl-tRNA (aa-tRNA) at the A site of the ribosome and therefore prevent the elongation phase during protein synthesis (40). Resistance to tetracyclines can arise from a variety of mechanisms: efflux, enzymatic degradation of the drug, protection of the ribosome binding site, and mutation in 16S rDNA (41). In *C. coli* and *C. jejuni*, tetracycline resistance genes are located on self-transferable plasmids. These genes have been described as genes that provide ribosome protection and have been designated tet (O) (42). They are present in a large number of *Campylobacter* isolates, obtained from different animal species (43, 44). They encode ribosomal protection proteins (RPPs) (40). Tetracyclines bind to ribosomes at the apex of site A, which in turn sterically blocks the aa-tRNA binding site and inhibits protein synthesis. When bound to tetracycline, GTP-bound RPPs will associate with the ribosome, leading to the release of tetracycline from site A. After tetracycline release, GTP is hydrolyzed and RPP dissociates from the ribosome, establishing protein synthesis. So far, tet (O) is the only *tet* gene currently identified in *Campylobacter* and confers resistance to the tetracycline class of antibiotics (6, 45). The presence of tet (O) in various Gram-positive bacteria (46) indicates its origin and exchange between species. In *C. jejuni*, tet (O) was first cloned from the transferable plasmid pUA466 (47). Sequencing of the two tetracycline resistance plasmids, one from strain *C. jejuni* 81–176 (48) and the other from strain *C. coli* CC31, revealed a large number of identical sequences and similar genome organization, despite the temporal and spatial distance of these two strains (49). Although the tet (O) gene is encoded by plasmids in most strains, in *Campylobacter* it can also be located on a chromosome. This phenomenon was demonstrated in 33% of tetracycline-resistant strains of *C. jejuni* originating from Alberta, Canada (50) and 76% of tetracycline-resistant isolates from Australia (51). The presence of an insertion element, IS607, was described on the tet (O) plasmid (50), and it is, therefore, possible that mobile genetic elements, other than transmissible plasmids, may be involved in the acquisition and spread of tetracycline resistance (8).

Tetracycline resistance in *C. jejuni* was associated with CmeABC efflux pump activity against a number of drugs (52).

### Mechanisms of Aminoglycoside Resistance

Aminoglycoside resistance genes are present in many bacterial species and encode enzymes that modify these antibiotics (53). These enzymes, based on the catalyzed reaction, can be divided into three different groups (aminoglycoside-phosphotransferase, aminoglycoside adenyl transferase, and aminoglycoside-acetyltransferase); however, all three groups act by a similar mechanism: they lead to the formation of 30-O-aminoglycoside phosphotransferase (13, 54). Aminoglycoside phosphotransferases are very prevalent in nature and are described in both Gram-positive and Gram-negative bacteria (55). *Campylobacter* may possess an apha-1 gene, located on a chromosome similar to that isolated from E. coli, indicating that this gene is from the Enterobacteriaceae family (56). In contrast, the apha-3 gene, previously found in Gram-positive cocci (57), was identified on plasmid pIP1433 in *C. coli* (54) and on the large plasmid in strains *C. jejuni* (50). More recently, plasmid *C. jejuni*, 25.7 kb in size, pCG8245, was described, and it contains sequences encoding copies of enzymes for inactivating aminoglycosides derived from Gram-negative and Gram-positive bacteria (58). The third resistance gene, the phosphotransferase gene, apha-7, was also identified on plasmid *C. jejuni* pS1178 (55). Although based on its sequences, 55% identity with this gene was detected in streptococci, the ratio of % G + C of 32.8% corresponds to the content of the chromosome *C. jejuni*, which indicates that the apha-7 gene may be endemic to the genus *Campylobacter* (55). Kanamycin resistance is often determined by a plasmid that also encodes tetracycline resistance (59), and this resistance can be transferred along with tetracycline resistance, a conjugation process, from a particular strain of *C. jejuni* to recipient *C. jejuni* (60).

### Integrons

Integrons, which carry transposons, are a major mode of spreading multiple antibiotic resistance (61) and are highly prevalent in Gram-negative intestinal bacteria found in animals (62). Integrons are the genetic structures of bacteria, which express and can acquire and exchange “gene cassettes.” Typically, these cassettes carry a single gene without a promoter. A nearby promoter is in charge of transcribing the entire series of tapes. It is considered that gene cassettes may be incorporated and excised via circular intermediates. In this way, recombinations between short sequences found at their ends, which are known as base elements 59 (59-be), and which do not have to be 59 bases long, would be included. The 59-be are a diverse family of sequences that functions as integrase-recognizing sites (enzymes responsible for integrating a gene cassette into an integron) that are site-specific (63). The roles of class 1 integrons and gene cassettes in the acquisition and spread of antibiotic resistance are well-known, so that over 75 gene cassettes carrying genes encoding resistance are described (64). Integral-like structures found in *Campylobacter spp*. may contribute to sulphonamide resistance (65). A class 1 integron associated with resistance to tobramycin and gentamicin, due to the existence of the aminoglycoside resistance gene, aacA4, was demonstrated in 5% of *C. jejuni* isolates derived from broiler chickens (62). In a large study of unrelated Irish thermophilic *Campylobacter*, 16% of isolates, both *C. jejuni* and *C. coli*, possessed complete integrin class 1 with a recombinant gene cassette containing the aminoglycoside resistance gene, aadA2 (66). A study by Zhao et al. (67) demonstrated that several new aminoglycoside resistance genes underlay the recent emergence of gentamicin-resistant *Campylobacter* in humans.

### Mechanisms of Resistance to β-Lactam Antibiotics

Mechanisms of resistance to β-lactam antibiotics, such as ampicillin and some extended-spectrum cephalosporins, are variable and not yet fully defined (68–70). With the exception of carbapenems (imipenem and meropenem), most strains of *C. jejuni* and *C. coli* are resistant to a large number of β-lactam antibiotics (13, 71, 72). Strains *C. jejuni* and *C. coli* show innate resistance to penicillin G and narrow-spectrum cephalosporins, which is associated with poor binding of these antibiotics to PBP (70). There are other mechanisms of acquired resistance, such as the production of β-lactamases (59, 68). Class D genes for β-lactamase were recently demonstrated in *C. jejuni* strains isolated in Australia (73). This gene is thought to be responsible for resistance to ampicillin, piperacillin, and carbenicillin in *C. jejuni* strains that are sensitive to β-lactam antibiotics (8). Taylor et al. (74) first suggested that the gene encoding β-lactamase in *Campylobacter* was located on a chromosome, since ampicillin resistance could not be transmitted by conjugation, simultaneously with tetracycline resistance. In the genome of *C. jejuni* NCTC 11168, a 774 bp gene was located that probably encodes periplasmic β-lactamase class D, Cj0299, 257 amino acids long (75). The corresponding gene derived from the human clinical isolate *C. jejuni* GC015 was cloned, described, and found to lead to the resistance to ampicillin, penicillin, and carbenicillin (73). This β-lactamase, designated OXA-61, has been shown to be identical to other OXA-type enzymes in *P. aeruginosa, Acinetobacter baumannii*, and *Fusobacterium*, and mediates resistance to penicillin, oxacillin, ampicillin, amoxicillin-clavulanate, piperacillin, and carbenicillin (72). The OXA-61 orthologous enzymes are present in *C. jejuni* RM1221 and *C. lari* RM2100 and are 99 and 52% identical in protein composition, respectively, to Cj0299 (http://campy.bham.ac.uk/). However, *C. jejuni* may produce more than one type of β-lactamase. Four enzymes are described based on their different activities according to 8 β-lactams, relative hydrolysis rate, molecular weight, immunological specificity, and isoelectric point (pI). The most commonly found beta-lactamase was called type A, had a size of 30 kDa with pI = 8.3, and was active against penicillin, ampicillin, oxacillin, and carbenicillin, weakly active against cephalothin, and did not act on cephaloridine, cefuroxime, and cefotaxime (76). β-lactamase derived from *C. jejuni* was shown to hydrolyze ampicillin, amoxicillin, penicillin and cloxacillin, and to partially hydrolyze cephalothin (77). The β-lactamase profile was similar to that of type A enzyme described by Lucain et al. (76), had a pI = 8.8 and could be inhibited by tazobactam, clavulanic acid, sulbactam, and cefoxitin, but not ethylene diamino tetra acetic acid (ethylene diamino tetra acetic acid, EDTA) or p-chloromercuribenzoate. Sequencing of the ten genomes of *Campylobacter* showed that metallo-β-lactamase production is also possible in these bacteria. However, two possible zinc hydrolases, structurally similar to the metallo-β-lactamase family in *C. jejuni*, did not lead to β-lactam resistance. Also, no β-lactamase activity was observed in *E. coli* or *C. coli* in which the zinc hydrolase gene was transformed, indicating that the enzyme does not function as a metallo-β-lactamase (8). Some *C. jejuni* isolates that do not possess OXA-61 produce β-lactamase CjBla2 which has pI = 9.2 and a molecular mass of 32.4 kD, which was confirmed by mass spectrophotometry. OXA-61, which dominates in *Campylobacter spp*. of animal origin (78), is similar to the β-lactamases described in isolates of human origin. The formation of OXA-61 is associated with resistance to foams but not cephalosporins. The combination of amoxicillin and clavulanic acid is considered to be effective against all tested isolates resistant to these antibiotics (79).

### Mechanisms of Sulphonamide Resistance

Resistance to sulphonamides in Gram-negative bacteria is usually due to the acquisition of drug-resistant variants of DHPS, which are transmitted horizontally (80). In Gram-positive bacteria, the most common mechanism is a mutation in the gene encoding DHPS (81, 82). Sulphonamide resistance in *C. jejuni* was found to be associated with mutation due to the substitution of four amino acid residues in DHPS leading to the reduced affinity for sulphonamides (83). Sulphonamides compete with PABA for DHPS, preventing PABA from incorporating into folic acid. Other mechanisms of sulphonamide resistance have not been described in *Campylobacter* so far (13).

### Mechanisms of Resistance to Trimethoprim

Trimethoprim acts by binding to and inhibiting dihydrofolate reductase (DFR) activity (13). Resistance is a consequence of the acquisition of dfr genes that are transferred horizontally and whose products are not inhibited by trimethoprim. In *Campylobacter*, two different genes (dfr1 and dfr9) have been described, which are responsible for the development of resistance. These genes are found on a chromosome in the transposon or integron. These two resistant dihydrofolate reductases have also been found in other Gram-negative bacteria, mainly members of the Enterobacteriaceae family, suggesting that *Campylobacter* may acquire genes responsible for trimethoprim resistance (72, 84, 85).

## Occurrence of Multiple Resistance

Alarming percentages of multidrug resistance of Campylobacter strains were observed in many countries (86–89) Resistance to several groups of antibiotics in *C. jejuni* may be the result of the existence of self-transferable plasmids or the action of an efflux mechanism (90–92). The energy-dependent, broad-specific efflux system is thought to be responsible for the development of multiple resistance (resistance to β-lactams, erythromycin, tetracycline, chloramphenicol, and quinolones) in the laboratory mutant of *C. jejuni* (21, 93). *C. jejuni* efflux pumps describes an efflux system for a number of drugs called CmeABC (22, 94). This system encodes an operon of three genes on the bacterial chromosome and consists of a transport protein, CmeB, which belongs to the resistance nodulation cell division (RND) family, then the periplasmic membrane fusion protein (CmeA) and outer membrane factor (CmeC). CmeABC is constitutively expressed in non-mutated types of *Campylobacter* and removes antibiotics (fluoroquinolones, erythromycin, tetracycline, chloramphenicol, and ampicillin), detergents, dyes (ethidium bromide), bile salts, and heavy metals (22). This efflux system is essential for the existence of innate antibiotic resistance and is necessary for resistance to fluoroquinolones. In doing so, CmeABC is necessary for the colonization of *Campylobacter, in vivo*, by allowing the bacterium to be resistant to bile, which is otherwise present in the digestive tract of mammals (95). CmeABC is controlled by the action of the CmeR transcription repressor when it encodes a gene located just above cmeABC. CmeR binds to the region of the cmeABC promoter and inhibits the expression of the efflux operon. The operon cmeABC is highly prevalent in strains of both *C. jejuni* and *C. coli* and is constitutively expressed in unmutated strains (12). Genome sequencing of *C. jejuni* NCTC 1116813 revealed the presence of a number of possible efflux pumps in *Campylobacter* (96). However, their function is yet unknown. In addition to CmeABC, *C. jejuni* also has another RND-type efflux pump, called CmeDEF (96), in which CmeD is probably the outer membrane channel protein, CmeE–periplasmic fusion protein, and CmeF–inner membrane conveyor. The role of CmeDEF in antibiotic resistance is still controversial (15), perhaps due to different findings of the authors and possible masking by the action of the CmeABC pump. It is thought to lead to multiple resistance, but also to transport ciprofloxacin (97). Although the level of CmeDEF expression is low, it acts together with CmeABC leading to the emergence of resistance to antibiotics and toxic compounds and probably acts as a secondary efflux mechanism (98). Inactivation of CmeF alone, in several strains of *C. jejuni*, increased the efficiency of the efflux mechanisms of mutants, indicating that mutation in CmeF increases the regulation of other efflux transporters. This finding indicates that, in the process of resistance development, there are very complex interactions of efflux transporters in *Campylobacter*. Both CmeDEF and CmeABC are important for the life support of *C. jejuni* (98). In recent years many researchers have reported novel multidrug resistance mechanisms in *Campylobacter* (99, 100).

## Congenital Resistance

*Campylobacter* shows innate resistance to various antibiotics such as cephalosporins (70), bacitracin, novobiocin, rifampin, streptogramin B, trimethoprim, and vancomycin (59, 101). Although the mechanisms of innate resistance are not clear enough, they are probably a consequence, at least in part, of the poor permeability of *Campylobacter* casings, as well as of the active efflux for which efflux pumps for a number of drugs are responsible (102).

## Significance of Resistant Strains

### Possible Influence of Infection With Resistant Strains of *Campylobacter* on the Severity of the Clinical Picture

Several epidemiological studies have examined the clinical impact of antibiotic resistance to *Campylobacter* infection. The patients, with isolated *C.jejuni* resistant to quinolones in Minnesota in 1997 had a mean duration of diarrhea of 10 days, compared with 7 days, in those in whom susceptible strains were isolated (103). In a retrospective study (104) comparing cases, five of the 28 patients (31%) in whom ciprofloxacin-resistant *Campylobacter* was detected, were hospitalized, compared with one in 31 patients (3%) with isolated susceptible strains. However, the median age of these two groups of patients was 46 and 24, respectively.

A case-control study conducted in the period 1998–1999 by the CDC showed that in a group of 290 individuals with ciprofloxacin-resistant *Campylobacter* infection who were not treated with antidiarrheals, the median duration of diarrhea was 9 days, compared with 7 days in patients with sensitive isolates (105). In 85 subjects who used fluoroquinolones alone, the median duration of diarrhea was 8 days in patients in whom resistant strains were isolated, and 6 days in those in whom susceptible isolates were detected. In 63 patients who did not use antibiotics, diarrhea caused by ciprofloxacin-resistant strains lasted an average of 12 days, as opposed to diarrhea caused by susceptible strains that lasted 6 days. In that study, patients with both susceptible and resistant strains were equally likely to be hospitalized. It was observed that persons in whom susceptible strains were isolated spent a longer time in the hospital (on average 3 days), compared to those with resistant ones (on average 2 days). However, one group of authors argues that when it comes to infections in the United States, no association has been established between antibiotic resistance and longer disease duration (106).

A study conducted in Denmark, from 2001 to 2002. Engberg et al. (107) showed that individuals in whom fluoroquinolone-resistant *C. jejuni* was isolated had a median disease duration of 13.2 days, compared with 10.3 days, in individuals infected with susceptible strains. It was not determined how many of these people used quinolones. *C. coli* was not found to have a difference in disease duration in individuals in whom susceptible and resistant strains were isolated. Another, also Danish study (108), examined the relationship between infection with susceptible and resistant *Campylobacter* strains and the occurrence of health side effects defined as invasive disease or death within 90 days. These effects were found to occur in 0.6% of patients. Patients with quinolone-resistant strains infected in Denmark had a significantly higher risk of side effects within 30 days (patients were grouped by sex, age, and comorbidity). Infection with erythromycin-resistant strains was also associated with an increased risk of side effects within 90 days.

However, a meta-analysis of these studies showed that the harmful effects were not caused by resistant strains, that the patients were elderly (aged 67–91 years), and that almost all of them had a severe concomitant disease. It was also found that the association between macrolide-resistant strain infection and adverse effects did not occur earlier (before 90 days) and it was not considered whether the patients were exposed to macrolides due to an underlying disease, such as respiratory disease (109, 110).

A meta-analysis conducted by Wassenaar et al. to test the hypothesis that *Campylobacter* resistant to fluoroquinolones gives a more severe clinical picture showed that there was no significant difference in the duration of the disease if caused by strains sensitive or resistant to fluoroquinolones. A longer duration of the disease occurred only if the infection occurred while traveling abroad (110).

Examination of the effect of fluoroquinolone resistance on the severity of the clinical picture in Finns showed that resistance to ciprofloxacin was not associated with particularly severe infections. In contrast, *Campylobacter* strains susceptible to ciprofloxacin, compared with resistant isolates, showed a tendency to cause more severe infections that were accompanied by bloody stools and required hospitalization (111).

### Possible Errors and Consequences for Human Health During the Treatment of *Campylobacter* Resistant to Macrolides

Only a small number of patients with campylobacteriosis are known to receive erythromycin, although this is the drug of choice (109). Erythromycin is given in diagnosed cases of campylobacteriosis, and most patients with diarrhea who seek medical help are treated empirically with broad-spectrum antibiotics. According to the results of a study conducted in Denmark, which included 122 patients with a known history of treatment, 40 (32.8%) were treated with antibiotics. Of these, 33 (82.5%) were treated with fluoroquinolones, 6 (15%) were treated with macrolides, and one (2.5%) was treated with both antibiotics (107). According to the results of other studies, <25% of treated cases of campylobacteriosis received erythromycin (103, 112).

Erythromycin was shown to shorten the duration of diarrhea in these patients if given immediately after the onset of symptoms (113). Therefore, patients who have experienced a treatment error due to macrolide-resistant *Campylobacter* may have a longer disease duration than those who have been successfully treated. However, in numerous studies, no error in therapy due to erythromycin resistance has been proven (103, 107). It is possible that the data are incomplete because the researchers are only looking for errors in the treatment of fluoroquinolone-resistant *Campylobacter*, or there are few patients infected with macrolide-resistant strains treated with erythromycin for analysis. Also, the outcome of treatment does not have to differ in patients in whom erythromycin-treated macrolide-resistant strains have been isolated and in patients in whom sensitive erythromycin-treated strains have been isolated. Empirical therapy of patients with campylobacteriosis shortens the duration of diarrhea by 2 days (114), but the therapy after laboratory diagnosis, which is a much more likely scenario in practice, does not alleviate symptoms (115). A meta-analysis of controlled studies of antibiotic treatment (macrolides and fluoroquinolones) showed that the intestinal symptoms reduce to only 1.3 days during the early, so-called, empirical treatment, but the authors still declare that a “restrictive approach” should be applied when prescribing antibiotics to the patients with no complications, i.e., patients who do not fall into any risk group (116).

Effective antibiotic therapy should be used in those patients who are seriously ill. In the UK, bacteraemia occurs on average in 1.5 per 1,000 cases of campylobacteriosis (117), and affects both people without a previous illness and people with AIDS (118). Of all patients with campylobacteriosis, those who are seriously ill will be the least likely to be exposed to therapeutic error, due to the recommendation that hospitalized patients be tested for susceptibility to macrolides and fluoroquinolones. If they develop resistance to macrolides, bacteraemia could be successfully treated with other classes of antibiotics (119). On the other hand, some therapeutic guides also suggest the possibility of the cautious use of fluoroquinolones in children (120).

Postinfectious sequelae that occur in patients with campylobacteriosis localized to peripheral nervous tissue, such as GBS and MFS, can have a very severe clinical picture. Although *C. jejuni* is the initiator of the autoimmune process in a large number of patients and although in some individuals it can be excreted in the stool for a very long time, there are currently no prospective studies on the effect of antibiotic therapy on disease course and shortening (121).

When it comes to postinfectious sequelae with manifestations on the musculoskeletal system, such as Reiter's syndrome or reactive arthritis, they have been shown to occur more frequently in individuals in whom initial diarrhea lasted longer (122). However, it is known that successful antibiotic treatment of the initial *Campylobacter* infection, as well as the shorter duration of diarrhea, do not prevent the development of Reiter's syndrome (123).

Given that *C. coli* is more resistant to macrolides, previous studies on its, perhaps, higher virulence compared to *C. jejuni* should also be considered (124). However, recent studies have shown that individuals infected with *C. jejuni* and *C. coli* have similar symptoms (107). Genome analysis has shown that possible virulence genes vary very little between these two species (125). It has also been shown that persons infected with macrolide-resistant *C. coli* do not have a more severe clinical picture than those infected with susceptible strains (108).

In the study of Elhadidy et al. (34), low resistance rates to streptomycin (4.5%) and erythromycin (2%) reflected the infrequent use of these antimicrobials in clinical settings. Moreover, these results promote the use of these antimicrobials in some European countries as efficacious therapeutic agents in health care settings in lieu of other antimicrobials against which *C*. *jejuni* has demonstrated increased resistance, including quinolones and fluoroquinolones.

### Persistence and Fitness of Antibiotic-Resistant *Campylobacter*

Mutations or other mechanisms that lead to the emergence of resistance in bacteria can affect their physiology (e.g., growth rate) having as a consequence their ability to adapt to an environment free of antibiotics.

In the absence of selective antibiotic pressure, *Campylobacter spp*. antibiotic-resistant may or may not suffer damage to fitness. Whether *Campylobacter spp*. antibiotic resistance will persist depends on its ability to be transmitted between hosts and cope with strains that are sensitive to antibiotics. This competition determines whether *Campylobacter* spp. antibiotic-resistant will prevail in the population or its number will decline in an antibiotic-free environment (6, 126). Resistance to fluoroquinolones resulting from a mutation in the gyrA gene can be maintained stably in *Campylobacter* even in the absence of selective pressure (23). *Campylobacter spp*. resistant to fluoroquinolones, which carries the most common mutation in the gyrA gene, colonizes chickens permanently, without the loss of resistance phenotype and resistance-related mutations, and its fitness is maintained. Competition experiments have shown that resistant mutants overcome susceptible strains in chickens (23), indicating that fluoroquinolone-resistant mutants possess improved adaptability. This change in fitness is associated with the Tre-86-Ile mutation, and the reversal of the Tre-86-Ile mutation into a non-mutated type allele is associated with a loss of advantage of such strains in chickens (6).

Based on the results of laboratory tests and monitoring of resistance, it is possible to predict that it will be difficult to reduce the resistance rate once a high prevalence of resistance to fluoroquinolones is established. The question arises as to how the gyrA mutation, associated with resistance, affects the ability of the bacterial population to maintain and increase its numbers. Mutations in DNA gyrase, which lead to resistance, are known to alter gyrase enzyme activities and affect DNA twisting. For the time being researchers are determining whether the mutations are sufficient to affect the physiology of *Campylobacter* and its fitness (6).

*Campylobacter* mutants that show low to moderate resistance to erythromycin, and do not have a mutation in 23S rRNA, are not stable in culture or host and rapidly lose their phenotype of resistance in the absence of macrolides (36). However, macrolide-resistant mutants, which carry the mutation in 23S rRNA, are highly resistant to erythromycin and their resistance phenotype is stable. They may persist in chickens even in the absence of competition with sensitive individuals (36). Unlike fluoroquinolone-resistant *Campylobacter*, erythromycin-resistant mutants do not have the ability to maintain their bacterial population compared to unmutated strains. Erythromycin-resistant mutants, which carry A2074G or A2075G mutations in 23S rRNA, have been shown to be rapidly overcome by isogenic non-mutated types (6). This phenomenon indicates that, in the absence of antibiotics, mutations that lead to resistance to macrolides lead to poorer adaptation of *Campylobacter* to the natural host. Discontinuation of tylosin to improve pig growth has led to a significant reduction in the number of erythromycin-resistant strains of *C. coli* (127).

The tetracycline resistance that tet (O) leads to is dominant in *Campylobacter* worldwide. Although this gene is usually located on the plasmid, it can also be located on the chromosome. Recent studies have shown that tetracycline resistance in *Campylobacter* has been observed in both the organic and conventional animal production systems (9) so that the maintenance of tetracycline-resistant *Campylobacter* strains is most likely not a consequence of antibiotic selection, but of possible coevolution of the plasmid containing the tet (O) gene and *Campylobacter*, and the plasmid itself is not a burden to the host and its maintenance as a resistant population (6).

## Discussion

Antibiotic resistance in *Campylobacter* remains a challenge for food safety and public health. So far, several mechanisms of antibiotic resistance have been discovered. These data, with information obtained on the association of isolates, provide a better understanding of how antibiotic resistance develops and is maintained, and how these microorganisms are transmitted to new hosts. Globally, the rate of antibiotic resistance is beginning to increase toward several antibiotics, and a profile of multiple resistance is emerging. Due to the high prevalence of resistance to fluoroquinolones, they are no longer as effective in the treatment of campylobacteriosis in humans. It is necessary to investigate the factors that affect the transmission and maintenance of this resistance in *Campylobacter* in different environments and in different hosts. It is also necessary to examine how resistance to fluoroquinolones affects the fitness of *Campylobacter*. Newer fluoroquinolones are more active against ciprofloxacin-resistant *Campylobacter* strains and new treatment regimens are needed that prevent the selection of fluoroquinolone-resistant mutants.

Macrolides are still the most effective antibiotics for the treatment of *Campylobacter* infection, however, the tendency to increase resistance in *C. jejuni* and *C. coli*, in some regions, requires more careful use of these antibiotics. Although some mechanisms of resistance are known, further research is needed to understand how macrolide-resistant *Campylobacter* is selected by the action of selective antibiotic pressure. The application of genomics and proteomics is expected to provide new insights into the molecular mechanisms of macrolide resistance. A multi-drug efflux pump, CmeABC, plays an important role in the transmission of antibiotic resistance in *Campylobacter*, but the contribution of other efflux transporters to antibiotic resistance as well as their natural function in *Campylobacter* should be examined (6).

In the treatment of campylobacteriosis, in addition to macrolides, several other drugs that are still very effective can be used, such as carbapenems and gentamicin, although the limitation of gentamicin is its excessive application. On the other hand, multi-resistance has been observed in some strains of *Campylobacter*. However, unlike *Pseudomonas aeruginosa* and *Burkholderia cepacia*, in which multidrug resistance in certain isolates has reached the level of pan-resistance, *Campylobacter* strains have not yet reached that point of ecological success.

Humanity has a responsibility to treat both sick animals and sick people. Problems occur when diseased animals are colonized or infected with similar microorganisms that have the potential to cause disease in humans. The number of effective drugs that can be used to successfully treat infections in both humans and animals is limited, and antibiotic resistance is on the rise faster than new drugs are discovered and before they reach the market. Therefore, it is necessary to carefully decide on the methods of administration of antibiotics to obtain optimal effects in both humans and animals. Farmers, veterinarians, food producers, clinicians, pharmacists, and consumers, both internationally and locally, are involved in this process. Also, both locally and globally, surveillance needs to be strengthened and trends monitored and communicated, especially if an increase in the resistance rate is identified, so that appropriate controls and interventions can be implemented to limit *Campylobacter* resistance, block the emergence and transmission of resistance (43). Since the emergence of MDR *Campylobacter* strains is attributable to the widespread use of antibiotics in poultry and pig production, these findings recommend the more cautious use of critical antimicrobial agents in swine and poultry production (14).

## Author Contributions

All authors listed have made a substantial, direct and intellectual contribution to the work, and approved it for publication.

## Conflict of Interest

The authors declare that the research was conducted in the absence of any commercial or financial relationships that could be construed as a potential conflict of interest. The reviewer IP declared a shared affiliation with one of the authors, SA to the handling editor at time of review.

## Publisher's Note

All claims expressed in this article are solely those of the authors and do not necessarily represent those of their affiliated organizations, or those of the publisher, the editors and the reviewers. Any product that may be evaluated in this article, or claim that may be made by its manufacturer, is not guaranteed or endorsed by the publisher.
